# Gene Ontology Analysis for Drug Targets of the Whole Genome Transcriptome of Human Vascular Endothelial Cells in Response to Proinflammatory IL-1

**DOI:** 10.3389/fphar.2019.00414

**Published:** 2019-04-24

**Authors:** Tom Skaria, Esther Bachli, Gabriele Schoedon

**Affiliations:** ^1^Inflammation Research Unit, Division of Internal Medicine, University Hospital Zürich, Zurich, Switzerland; ^2^Department of Medicine, Uster Hospital, Uster, Switzerland

**Keywords:** inflammation, vascular endothelium, IL-1β, transcriptome profiling, drug targets

## Introduction

The innate immune system combats tissue injury and infection by activating the proinflammatory responses involving the humoral complement system, granulocytes, macrophages and vascular endothelial cells (VEC) (Newton and Dixit, 2012; Zhu et al., 2012). Macrophages mediate proinflammatory responses by releasing inflammatory cytokines such as IL-1β. Once secreted, IL-1β paracrinically acts on the VEC and massively change their functions. These perturbations include a change from the anticoagulant phenotype to a procoagulant state, enhanced expression of vasoactive substances, cell adhesion molecules as well as inflammatory mediators including chemoattractants, and endothelial barrier dysfunction causing microvascular leakage (Pober and Sessa, 2007). Although essential for the effective immune defense, uncontrolled or chronic inflammatory response causes tissue damage and loss of organ function (Lon et al., 2012).

Increased IL-1β expression and an aberrantly activated IL-1β signaling explicitly correlates with disease progression in a broad spectrum of local or systemic acute and chronic inflammatory diseases such as severe systemic inflammatory response syndrome, sepsis, inflammatory bowel disease and rheumatoid arthritis, and malignancies such as myeloma. In such diseases, blocking IL-1β signaling with naturally occurring IL-1 receptor (IL-1R) antagonist (IL-1Ra) Anakinra or neutralization with anti-IL-1β monoclonal antibody leads to abrupt and sustained decrease in disease severity (Dinarello, 2011). The classical IL-1β pathway involves the binding of IL-1β to IL-1R that results in the recruitment of the adapter protein MyD88 to the receptor's cytoplasmic domain. It is followed by the activation of IL-1R-associated kinases (IRAKs), and phosphorylation inactivation of inhibitor of κB (iκB). The inactivated iκB is then targeted for proteasomal degradation, thereby freeing NF-κB, the latter then translocates to the nucleus to turn on the transcription of genes involved in inflammatory responses (Liu and Malik, 2006; Dinarello, 2011). The standard approaches targeting IL-1β signaling by blocking its production or interactions with IL-1R may arrest the NF-κB pathway. When NF-κB-dependent immune responses critical for defense are interrupted, it renders the host immunocompromised and susceptible to infections (Keane et al., 2001; Tak and Firestein, 2001; Zhu et al., 2012). Therefore, strategies modulating IL-1β-mediated proinflammatory responses without affecting central NF-κB activation or targeting a specific cell population like vascular endothelium are on great demand. Further, recent evidences suggest that targeting a specific perturbation of IL-1β-activated VEC (for example, enhanced immune cell attachment or barrier dysfunction) may limit tissue destruction (McCulloch et al., 2006; Zhu et al., 2012).

In the present study, we employed transcriptome profiling to define the genes regulated by IL-1β signaling in the well-established model of adult human immunocompetent primary VEC, human coronary artery endothelial cells (HCAEC) (Zeuke et al., 2002; Franscini et al., 2004). Here, we identify HGF as one of the therapeutically relevant genes upregulated by IL-1β. The gene ontology analysis suggests HGF's critical involvement in one of the most significant VEC monolayer barrier-injuring biological pathways and inflammatory diseases enriched in IL-1β transcriptome in VEC.

## Materials and Methods

### Cell Culture

Human coronary artery endothelial cells (HCAEC) were propagated and treated with recombinant human IL-1β (20 U/mL, PeproTech) as described previously (Skaria et al., 2017a,b) and given in the Supplementary Methods. Detailed information on endothelial cell characterization is given in Supplementary Methods.

### Differential Gene Expression Profiling

Microarray based gene expression profiling, as well as scanning, feature extraction, and data normalization of microarrays were carried out as described previously (Skaria et al., 2016, 2017b) and given in the Supplementary Methods. Entire data sets of IL-1β-regulated transcriptome in HCAEC are accessible in the NCBI GEO data repository, accession numbers: GSE62281, GSE118297.

Microarray analysis using GeneSpring GX 9.0 Software (Agilent Tech. Inc.) and MetaCore™ GeneGO software (Thomson Reuters, http://portal.genego.com) was carried out as described previously (Skaria et al., 2016, 2017b) with modifications and given in the Supplementary Methods.

## Data Description

### Drug Targets in IL-1β-Regulated Transcriptome of Adult Human VEC

The transcriptome profile of 4 h IL-1β-treated HCAEC was compared to that of untreated HCAEC using whole human genome oligomicroarrays. The genes in treated cells that consistently exhibited a minimum two-fold change in expression compared with untreated cells in preprocessed transcriptome data were identified using GeneSpring analysis. IL-1β regulated 531 genes, of which 374 genes were upregulated and 157 genes were down regulated (Supplementary Table S1).

To identify the genes regulated by IL-1β in HCAEC that encode targets for known drugs and thus are of therapeutic relevance, genes regulated at least two-fold in their expression were subjected to drug target analysis using GeneGO software. IL-1β regulated the gene expression of 26 direct drug targets, of which 21 were upregulated (Supplementary Table S2) and 5 were down regulated (Supplementary Table S3). Among the regulated targets (Supplementary Tables S2, S3), the expression of genes such as COX-2, TNF-α, IL-6, IL-1β, MMP-1, GM-CSF, ETS-1, NF-kB1, and c-Rel have been reported being modulated in inflamed endothelium (Dinarello, 2011; Libby, 2017; Skaria et al., 2017b).

### Dysregulated HGF Expression Is Mapped to VEC Monolayer Barrier-Injuring and Repair Pathway

To our knowledge, the expression of genes such as *CTSS, HGF, PDE5A*, and *SRD5A2* (Supplementary Table S2) was not previously reported being regulated by IL-1β in adult human VEC. Therefore, we sought to investigate whether the regulated expression of these genes as found in the present study (Supplementary Tables S2, S3) critically modulates pathways involved in proinflammatory response to IL-1β in adult human VEC. To identify the most significant pathways regulated by IL-1β in HCAEC, all genes regulated at least two-fold in their expression were subjected to GeneGO functional enrichment analysis (EA). The “Glomerular injury in Lupus Nephritis,” “PDE4 regulation of cyto/chemokine expression in arthritis,” “Immune response-IL-17 signaling pathways,” and “Immune response-MIF-mediated glucocorticoid regulation” were the first, second, third and fourth most statistically significant pathways for IL-1β in HCAEC (Supplementary Figure S1). The genes of these top four pathways regulated by IL-1β (Supplementary Table S4) are the targets of NF-kB, the master regulator of immune responses, and comprise a spectrum of inflammatory cytokines (e.g., IL-1, IL-6), chemokines (e.g., CCL2, CCL5), intercellular adhesion molecules (e.g., ICAM, VCAM), matrix metalloproteinases (e.g., MMP-1), and prostaglandin (COX-2; Supplementary Table S4).

**Figure 1:**
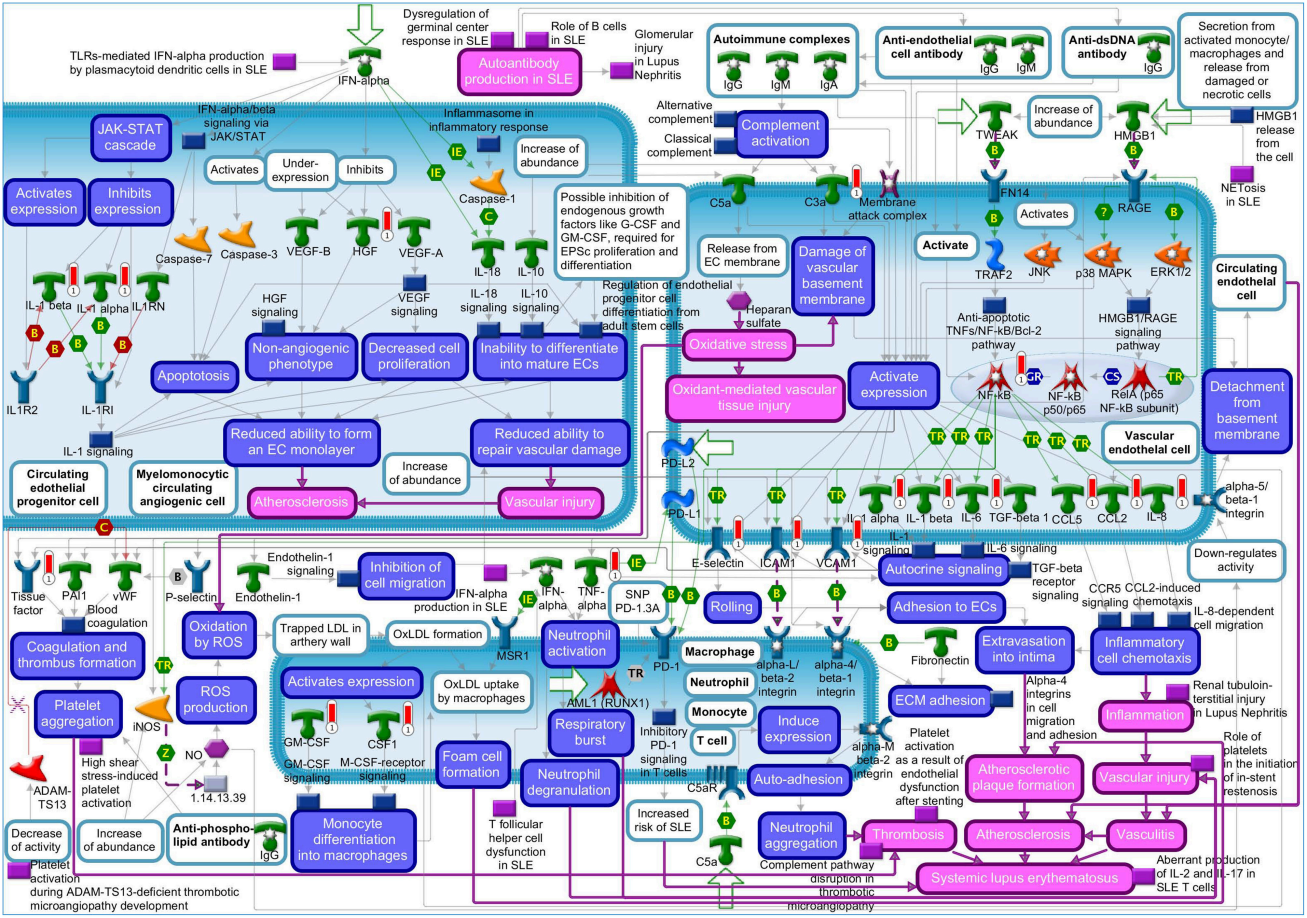
Metacore™ map showing the signaling context of the genes contained in the “Vascular endothelial cell damage in SLE” pathway. Genes upregulated by 4 h IL-1β treatment are marked by red thermometer icons. Data are from three independent experiments.

The “Vascular endothelial cell damage in SLE” was the fifth most significant pathway for IL-1β in HCAEC (Supplementary Figure S1). Among the genes regulated in this pathway are *HGF* and the proinflammatory target genes of NF-kB described above (Supplementary Table S4). The MetaCore™ map of this pathway showing the genes within their signaling context indicates that HGF regulates the monolayer forming properties of the endothelium (Figure 1, upper left quarter). The GO analyses listed the involvement of HGF in 128 disease states (Supplementary Tables S5, S6) and further revealed “Vascular endothelial cell damage in SLE” as one out of the total seven pathological pathway maps where HGF has an established role (Supplementary Table S7). The “Immune response_HMGB1/RAGE signaling pathway,” “Substance P-mediated inflammation and pain in Sickle cell disease,” “Immune response_IL-18 signaling,” “Signal transduction_NF-kB activation pathways,” and “Immune response_Alternative complement pathway” were the sixth, seventh, eighth, ninth, and tenth most significant pathways regulated by IL-1β in HCAEC (Supplementary Figure S1). The genes regulated by IL-1β in the latter pathway are involved in complement activity while those in the other four pathways are mainly the target genes of NF-kB associated with inflammatory responses involving endothelial activation as described already in the top four pathways (Supplementary Table S4).

Since IL-1β regulated HGF expression (Supplementary Tables S1, S2) and the regulated HGF expression has been mapped to one out of the 10 most significant pathological pathways regulated by IL-1β in HCAEC (Supplementary Figure S1: 5th pathway, Supplementary Table S4), we next checked if HGF is associated with the most significant disease profiles enriched in IL-1β transcriptome. GeneGO functional EA analysis via the biomarker assessment work flow revealed the critical involvement of HGF in “Lupus Erythematosus, Systemic,” the 5^th^ out of the ten most significant “Immune System Disease” enriched in IL-1β transcriptome (Supplementary Figure S2, Supplementary Table S8).

Targeting the inflammatory responses induced by the potent innate immune proinflammatory cytokine IL-1β in VEC has been found to reduce tissue damage and is emerging as a therapeutic strategy to prevent loss of organ function during local and systemic inflammatory diseases. Most often, studies aimed at evaluating the inflammatory responses in adult human vascular endothelial cells *in vitro* depend merely on cultured human umbilical vein endothelial cells (HUVEC). HUVEC derived from the immune naïve fetal tissue was subsequently reported to exhibit significant differences in function compared with adult human VEC and therefore may represent an inappropriate model of adult human vascular endothelium (O'donnell et al., 2000; Tan et al., 2004; Hwang et al., 2018). To study the proinflammatory effects of IL-1β on adult human primary VEC, we used primary endothelial cells isolated from adult human coronary artery, that were positively tested for vascular endothelial markers and function and are well-recognized as immunocompetent (Zeuke et al., 2002; Franscini et al., 2004; Skaria et al., 2017b). IL-1β treatments of HCAEC in this study were conducted for 4 h since IL-1β is well-established as an early response cytokine and capable of causing inflammatory gene induction and responses as early as 4 h (Mizgerd et al., 2001; Sadeghi et al., 2015; Skaria et al., 2017b). Here, we show that IL-1β upregulates the gene expression of *HGF, BDKRB2, CTSS*, and *SERT* that are critically involved in regulating VEC monolayer barrier function. The expression of these genes was not previously reported being regulated by IL-1β in adult human VEC. The functional enrichment analysis maps HGF's dysregulated expression to one of the most significant VEC monolayer barrier-injuring and repair pathways, and inflammatory diseases enriched in IL-1β transcriptome in VEC.

## Future Directions

Besides systemic lupus erythematosus, several acute systemic inflammatory diseases like systemic inflammatory response syndrome and sepsis show altered plasma levels of both HGF and IL-1β (Sakon et al., 1996; Matsushima et al., 2004; Sekine et al., 2004). In these disease states, VEC barrier breakdown and subsequent hyperpermeability leading to tissue edema represents a critical factor contributing to the morbidity and mortality (Weis, 2008; Chava et al., 2012). Therefore, the present finding that IL-1β induces HGF in VEC raises important questions whether (1) IL-1β-activated VEC represents a major source of increased HGF levels in IL-1β-associated inflammatory diseases, (2) HGF has a role in regulating IL-1β-induced VEC injury and dysfunction, (3) therapeutically targeting HGF exerts beneficial or deleterious effects on VEC barrier integrity and function in pathophysiological states. Similar studies should also be performed to evaluate the critical roles and benefits of therapeutic targeting of BDKRB2, CTSS, and SERT, which were previously found to contribute to VEC dysfunction and are found to be induced by IL-1β in human VEC in the present study.

## Author Contributions

TS, EB, and GS conceived and designed the research and wrote the manuscript. TS performed the experiments. TS and GS analyzed the data. All authors read and approved the final manuscript.

### Conflict of Interest Statement

The authors declare that the research was conducted in the absence of any commercial or financial relationships that could be construed as a potential conflict of interest.
