# Supplementary material for: Gene Ontology Analysis for Drug Targets of the Whole Genome Transcriptome of Human Vascular Endothelial Cells in Response to Proinflammatory IL-1
Source: Front Pharmacol. 2019 Apr 24;10:414. doi: 10.3389/fphar.2019.00414 (PMC6491677; doi:10.3389/fphar.2019.00414)

Supplementary Material

Gene Ontology Analysis for Drug Targets of the Whole Genome Transcriptome of Human Vascular Endothelial Cells in Response to Proinflammatory IL-1

Tom Skaria[1], Esther Bachli[2], Gabriele Schoedon[1*]

[1]Inflammation Research Unit, Division of Internal Medicine, University Hospital Zürich, Zürich, Switzerland, [2]Department of Medicine, Uster Hospital, Uster, Switzerland.

[*]**Correspondence**
Gabriele Schoedon
Email: klinsog@usz.uzh.ch

Supplementary Methods

Supplementary Results

Supplementary Table S1. Genes regulated (≥2 fold up or down) by 4 h IL-1β treatment in HCAEC. Data are from 3 independent array experiments.

Supplementary Table S2. Genes that encode direct targets for known drugs and are upregulated by IL-1β in HCAEC. Data are from 3 independent array experiments.

Supplementary Table S3. Genes that encode direct targets for known drugs and are downregulated by IL-1β in HCAEC. Data are from 3 independent array experiments.

Supplementary Table S4. The targets of most significant pathways regulated by 4 h IL-1β treatment in HCAEC. Data are from 3 independent array experiments.

Supplementary Table S5. GO listing of disease states in which either HGF or IL-1β are involved.

Supplementary Table S6. GO listing of disease states in which both HGF and IL-1β are involved.

Supplementary Table S7. GO listing of pathways associated with HGF.

Supplementary Table S8. Targets involved in 'Lupus Erythematosus, Systemic' that were regulated by IL-1β in HCAEC as listed by Metacore GO biomarker assessment work flow centering immune system diseases. Data are from 3 independent array experiments.

Supplementary Figure S1. The ten biological pathways most significantly ($P<0.05$) regulated by 4 h IL-1β treatment in HCAEC. Pathways represented as histograms are ranked by the –log value (*P* value). Data are from 3 independent experiments.

Supplementary Figure S2. Metacore™ GO biomarker assessment work flow centering immune system diseases showing the top 10 disease profiles enriched in 4 h IL-1β transcriptome. Diseases represented as histograms are ranked by the –log value (*P* value). Data are from 3 independent experiments.

Supplementary Methods

Cell culture

HCAEC (Cat. No. CC-2585) were propagated in EBM-2 medium (Clonetics, Lonza) added with EGM-2MV Single Quots and 5% FBS (Clonetics, Lonza) in a Class 100 HEPA air filtered system (SteriCult, Fisher Scientific, Switzerland) as described previously (Skaria et al., 2017b; Skaria et al., 2017a). HCAEC used in this study were tested positively and functionally for CD31 (PECAM-1), CD105 (endoglin), von Willebrand Factor VIII, and acetylated low-density lipoprotein uptake as certified by the manufacturer (Lonza, Cell Systems). To prevent masked low-level contamination in cultures, antibiotic-free culture medium was used during the whole study period. Experiments were conducted using only cells from passages 3- 6. HCAEC monolayers were treated with recombinant human IL-1β (20 U/mL, Cat. No. 200-01B, PeproTech) for 4 h. For all pipetting steps, TipOne aerosol barrier sterile filter pipet tips (USA Scientific) were used.

Differential gene expression profiling

Gene expression profiling through competitive two-colour hybridization of cRNA probes onto 4×44K Human Whole Genome Oligonucleotide microarrays (Agilent Technologies) was carried out as described previously (Schaer et al., 2013; Skaria et al., 2016; Skaria et al., 2017b). After total RNA was isolated using RNeasy Mini Kit (Qiagen, Basel, Switzerland), it was then quantified by Nanodrop spectrophotometry. RNA integrity was verified using the Agilent 2100 Bioanalyzer System (Agilent Tech., Basel, Switzerland). Microarray experiments were conducted using only RNA samples that showed a RNA integrity number (RIN) >9. 500 ng of total RNA from every sample was reverse transcribed and labelled with Cy3- and Cy5-CTP utilizing the two-colour Quick Amp Labelling Kit (Agilent Tech., Switzerland) consisting of internal control probes and spike in's (labelled Spike A mix with Cy-3 and Spike B mix with Cy-5) for control of reaction performance and background normalization of arrays. cRNA fragmentation and hybridization onto the Human GE 4×44K V2 Microarray chips were carried out according to the Quick Amp Labelling protocol (Agilent Techno., version 5.7, 2008). To prevent ozone-induced deterioration of cyanine dyes, array chips were washed with stabilization and drying solution (Agilent Tech., Switzerland). Scanning, feature extraction and pre-processing along with data normalization of the microarrays were carried out using the Microarray Scanner and Feature Extraction Software 10.7 (Agilent Tech. Inc.) with default settings for Agilent 4×44 K two-colour arrays. Spot values were normalized using the default linear-lowess normalization. The pre-processed data was further analysed using GeneSpring GX 9.0 Software (Agilent Tech. Inc.) with default settings for two-colour arrays to select genes that were consistently regulated ≥ 2-fold in their expression in three independent microarray experiments (Supplementary Table S1). Gene ontology analysis was performed using Metacore GeneGO software version 6.32.69020 (Thomson Reuters, http://portal.genego.com). Genes regulated ≥2-fold in their expression as found from GeneSpring analysis and satisfying a P value < 0.05 and FDR cut off (0.05) were grouped into pathways according to their biological functions and gene ontology classes.

References

Schaer, C.A., Deuel, J.W., Bittermann, A.G., Rubio, I.G., Schoedon, G., Spahn, D.R., Wepf, R.A., Vallelian, F., and Schaer, D.J. (2013). Mechanisms of haptoglobin protection against hemoglobin peroxidation triggered endothelial damage. *Cell Death and Differentiation* 20**,** 1569-1579.

Skaria, T., Bachli, E., and Schoedon, G. (2017a). WIF1 prevents Wnt5A mediated LIMK/CFL phosphorylation and adherens junction disruption in human vascular endothelial cells. *Journal of Inflammation (London, England)* 14**,** 10.
Skaria, T., Bachli, E., and Schoedon, G. (2017b). Wnt5A/Ryk signaling critically affects barrier function in human vascular endothelial cells. *Cell Adh Migr* 11**,** 24-38.
Skaria, T., Burgener, J., Bachli, E., and Schoedon, G. (2016). IL-4 Causes Hyperpermeability of Vascular Endothelial Cells through Wnt5A Signaling. *PLOS ONE* 11**,** e0156002.

Supplementary Results

Supplementary Table S1. Genes regulated (≥ 2-fold up or down) by 4 h IL-1β treatment in HCAEC. Data are from 3 independent array experiments.

Upregulated genes	Downregulated genes
ABCA1	*ACOT6*
ABTB2	*ACSM5*
ACOXL	*AKAP5*
ADAMTS9	*ANKRD23*
ADAP1	*ARHGAP18*
AMPD3	*ATXN3L*
APOBEC3G	*BIN2*
APOL2	*C10orf107*
APOL3	*C10orf68*
APOL4	*C12orf54*
APOL6	*C13orf15*
ARID5B	*C18orf34*
ATF3	*C1orf192*
ATP2C2	*C1orf229*
BATF3	*C6orf10*
BCL2A1	*C9orf66*
BCL6	*CAGE1*
BCO2	*CAMKMT*
BDKRB2	*CAPS2*
BHLHE41	*CCDC148*
BICC1	*CCDC88B*
BIRC3	*CCNC*
BMP2	*CD200R1*
BSND	*CD37*
BTBD9	*CD5*
BTG3	*CENPN*
C10orf93	*CES1P1*
C11orf44	*CLEC1B*
C11orf96	*CLEC4GP1*
C12orf50	*CRYAA*
C15orf48	*CXCR4*
C1orf172	*DAPL1*
C1QTNF1	*DDIT4L*
C1R	*DIS3L2*
C1S	*DNAJB4*
C22orf33	*DYNLRB2*
C22orf41	*E2F2*
C2CD4A	*EGFL7*
C2CD4B	*EHD3*
C3	*EMX2*
C5orf49	*ETV1*
C5orf56	*FAM124B*
C6	*FAM201A*
C6orf58	*FAM26D*
C8orf4	*FAM53C*

CBLN4	*FAM69A*
CBR3	*FAM74A1*
CCL1	*FCRL3*
CCL11	*FGFR2*
CCL2	*FILIP1*
CCL20	*FLJ25328*
CCL3	*FMN2*
CCL4	*FOXP3*
CCL5	*FRY*
CCL7	*GATA4*
CCL8	*GFOD1*
CCRN4L	*GLYAT*
CD69	*GPR87*
CD70	*HOXA11-AS1*
CD83	*HOXA7*
CFB	*HOXC5*
CHDH	*HS3ST1*
CHST6	*HS6ST3*
CITED4	*HTR1D*
CLDN1	*IL16*
CLDN14	*KAZALD1*
CNTN2	*KHK*
CSF1	*KIF2B*
CSF2	*LOC100128164*
CSF3	*LOC100129518*
CTHRC1	*LOC100130157*
CTSS	*LOC100130255*
CX3CL1	*LOC100130452*
CXCL1	*LOC100130876*
CXCL10	*LOC100131089*
CXCL2	*LOC100131581*
CXCL3	*LOC158376*
CXCL5	*LOC282980*
CXCL6	*LOC284080*
CXCR7	*LOC286068*
CYLD	*LOC286071*
CYP1A1	*LOC401847*
CYP1B1	*LOC440117*
DDX58	*LOC644662*
DENND2D	*LOC646034*
DLG2	*LOC647163*
DNAH5	*LRRC8B*
DNAJB9	*LYL1*
DNM1L	*LYPD1*
DRAM1	*MARVELD3*
DUSP16	*MBL1P*
EBI3	*MCHR2*
EDNRA	*MDFI*
EFCAB9	*MICU1*
EFNA1	*MTUS1*

EGFL6	*MUC12*
EGR2	*MYCN*
EGR3	*NCRNA00307*
EGR4	*NCRNA00320*
ELOVL7	*NEB*
EPB41	*NPIPL3*
ERAP1	*NUAK1*
ETS1	*ODF2L*
ETV3	*OR5B21*
ETV7	*OR9G4*
F3	*OTX1*
FAM101A	*PCBP3*
FAM122C	*PCDH15*
FAM129A	*PDE1C*
FAM150B	*PHTF2*
FAM155B	*PLB1*
FAM201B	*PNMAL1*
FBXO48	*PPFIBP2*
FCRL2	*PPM1H*
FGF18	*PRICKLE1*
FGF5	*PRO0611*
FILIP1L	*PRPS1*
FLJ10038	*PSG9*
FLJ31104	*RAD9B*
FLJ44674	*RAET1E*
FOXF1	*RAG1*
FOXJ1	*RARB*
FSTL3	*RASSF9*
G0S2	*RD3*
GABRQ	*RIN1*
GBP1	*RNASE10*
GBP2	*RPL31P11*
GBP3	*SCD5*
GBP4	*SCNN1A*
GBP5	*SEMG1*
GCH1	*SERTAD4*
GFPT2	*SEZ6L*
GGTA1P	*SFRP2*
GJD3	*SFTPB*
GOT1L1	*SH3TC2*
GPR37L1	*SIRPD*
GPR68	*SNORA2B*
GRAMD3	*SOX18*
GVINP1	*SSTR1*
GXYLT2	*ST8SIA4*
GYPE	*TBX1*
HAS3	*TEX15*
HEY1	*THBD*
HGF	*TMEM100*
HIGD2B	*TMEM121*

HIVEP2	*TMEM41A*
HOMER1	*TPD52L3*
HOXB9	*TRIM45*
HS3ST3B1	*TRIOBP*
HTR3E	*TTC16*
ICAM1	*USH2A*
ICAM4	*YIF1B*
ICOSLG	*ZNF233*
IDO1	*ZNF253*
IER3	*ZNF30*
IFI30	*ZNF367*
IFIH1	*ZNF395*
IFIT5	
IFNGR2	
IL15	
IL15RA	
IL18R1	
IL18RAP	
IL1A	
IL1B	
IL36G	
IL6	
IL7R	
IL8	
INHBA	
IQCA1	
IRAK2	
IRF1	
IRX2	
ISG20	
JAM2	
KBTBD8	
KCNN2	
KDM6B	
KIAA1199	
KIRREL2	
KLF6	
KLK10	
KRIT1	
KYNU	
LAD1	
LAMB3	
LAMC2	
LIF	
LITAF	
LOC100128644	
LOC100128729	
LOC100129104	
LOC100130921	
LOC100131015	

LOC100289255
LOC100505599
LOC100507410
LOC144817
LOC152024
LOC283352
LOC284570
LOC285972
LOC339240
LOC389300
LOC440896
LOC644366
LOC728228
LOC729444
LTB
LYPD6
MAP3K8
MBP
MIR155HG
MMP1
MMP10
MS4A2
MSC
MSX1
MTHFD2L
MTMR7
MYB
MYO3A
NAB1
NAG20
NAMPT
NCOA7
NEURL3
NFKB1
NFKBIA
NFKBID
NFKBIZ
NINJ1
NIPAL4
NKD1
NKD2
NKX3-1
NKX3-1
NNMT
NOD2
NOV
NR4A3
NR6A1
NRIP1
NRXN1

NTN1
NUAK2
NUPL1
ODZ1
OOEP
OR11H12
OR5P2
OSGIN2
OXTR
P2RY6
PARD3
PARP14
PDE4DIP
PDE5A
PDGFRA
PDLIM4
PDZD2
PDZD4
PEG10
PELI1
PIM1
PION
PITPNC1
PITX2
PPAP2B
PRINS
PRRX1
PSMB11
PTGFR
PTGS2
PTPRC
PTX3
RASGRP1
RCAN1
RCSD1
REL
RELB
RFPL4B
RGS16
RIPK2
RND1
RRAD
RSPO3
RTP4
S100A14
S100A3
SAMD4A
SAMD9L
SAT1
SAV1

SCARNA23
SDC4
SELE
SEMA3C
SERPINA3
SERPINB13
SERPINB2
SERPINE2
SETMAR
SF3B2
SGPP2
SLC10A7
SLC12A7
SLC19A2
SLC2A6
SLC32A1
SLC41A2
SLC44A3
SLC45A2
SLC4A5
SLC6A4
SLC7A2
SLCO4A1
SMPX
SNORD12B
SOCS1
SOD2
SPON2
SQRDL
SRD5A2
ST6GAL1
STAT5A
STC2
STON2
SYT9
TAP1
TCHH
TDRD6
TFAP2A
TIAM2
TIFA
TLE1
TLR2
TMCO2
TMEM106A
TMEM217
TMEM71
TMPRSS9
TNF
TNFAIP2

TNFAIP3
TNFAIP6
TNFAIP8
TNFRSF11B
TNFSF10
TNFSF9
TNIP1
TNIP3
TPPP
TRAF1
TRAM1L1
TRAPPC3
TRIM36
TRIM58
TTC39A
UBD
UGCG
UMODL1
UPP1
VCAM1
VSTM1
WTAP
ZBTB10
ZC3H12A
ZC3H12C
ZNF140
ZNF391
ZNF599
ZNF664-FAM101A

Supplementary Table S2. Genes that encode direct targets for known drugs and are upregulated by IL-1β in HCAEC. Data are from 3 independent array experiments.

Gene symbol	Target	Type	Drug		Effect
PTGS2	COX-2 (PTGS2)	Enzyme	1	Aceclofenac	Inhibition
			2	Acemetacin	Inhibition
			3	Apricoxib	Inhibition
			4	Aspirin	Inhibition
			5	Bromfenac	Inhibition
			6	Butibufen	Inhibition
			7	Celecoxib	Inhibition
			8	Curcumin	Inhibition
			9	Diflunisal	Inhibition
			10	Droxicam	Inhibition
			11	Etodolac	Inhibition
			12	Etoricoxib	Inhibition
			13	Fenbufen	Inhibition
			14	Fenflumizol	Inhibition
			15	Fenoprofen	Inhibition
			16	Ibufenac	Inhibition
			17	Ibuprofen	Inhibition
			18	Ketorolac	Inhibition
			19	Leflunomide	Inhibition
			20	Lornoxicam	Inhibition
			21	Loxoprofen	Inhibition
			22	Lumiracoxib	Inhibition
			23	Meclofenamic acid	Inhibition
			24	Meloxicam	Inhibition
			25	Mesalazine	Inhibition
			26	Metamizole	Inhibition
			27	Nabumetone	Inhibition
			28	NCX701	Inhibition
			29	Nitroaspirin	Inhibition
			30	Nitroflurbiprofen	Inhibition
			31	Parecoxib	Inhibition
			32	Parsalmide	Inhibition
			33	Phenacetin	Inhibition
			34	Piroxicam	Inhibition
			35	Rebamipide	Activation
			36	Rofecoxib	Inhibition
			37	Salsalate	Inhibition
			38	Sulindac	Inhibition
			39	Tarenflurbil	Inhibition
			40	Tenidap	Inhibition
			41	Tenoxicam	Inhibition
			42	Terbogrel	Inhibition

			43	Tiaprofenic acid	Inhibition
			44	Tilmacoxib	Inhibition
			45	Timegadine	Inhibition
			46	Tolfenamic acid	Inhibition
			47	Tolmetin	Inhibition
			48	Trisalicylate	Inhibition
			49	Valdecoxib	Inhibition
			50	Zaltoprofen	Inhibition
			51	Zomepirac	Inhibition
SLC6A4	SERT	Solute carrier	1	Amoxapine	Inhibition
			2	Bicifadine	Inhibition
			3	Clomipramine	Inhibition
			4	DOV216303	Inhibition
			5	Duloxetine	Inhibition
			6	Fluvoxamine	Inhibition
			7	Iometopane (123I)	Inhibition
			8	Minaprine	Inhibition
			9	Nefazodone	Inhibition
			10	NS2359	Inhibition
			11	Paroxetine	Inhibition
			12	Prazepine	Inhibition
			13	Sertraline	Inhibition
			14	Sibutramine	Inhibition
			15	Trazodone	Inhibition
			16	Venlafaxine	Inhibition
TNF	TNF-alpha	Receptor ligand	1	Adalimumab	Inhibition
			2	Aloperine	Inhibition
			3	AME527	Inhibition
			4	Calcitriol	Inhibition
			5	CB1093	Inhibition
			6	CDP571	Inhibition
			7	Certolizumab pegol	Inhibition
			8	Doxycycline	Inhibition
			9	Etanercept	Inhibition
			10	Genz-29155	Inhibition
			11	Golimumab	Inhibition
			12	Infliximab	Inhibition
			13	Lenalidomide	Inhibition
			14	Onercept	Inhibition
			15	Pegsunercept	Inhibition
			16	Thalidomide	Inhibition
EDNRA	EDNRA	Receptor	1	Ambrisentan	Inhibition
			2	Atrasentan	Inhibition
			3	Avosentan	Inhibition
			4	BMS-193884	Inhibition

			5	Darusentan	Inhibition
			6	Enrasentan	Inhibition
			7	Macitentan	Inhibition
			8	Nebentan	Inhibition
			9	Sitaxentan	Inhibition
			10	Sparsentan	Inhibition
			11	TBC3711	Inhibition
			12	Tezosentan	Inhibition
			13	Zibotentan	Inhibition
PDE5A	PDE5A	Enzyme	1	Avanafil	Inhibition
			2	Ibudilast	Inhibition
			3	OSI461	Inhibition
			4	Sildenafil	Inhibition
			5	SLx2101	Inhibition
			6	Tadalafil	Inhibition
			7	Udenafil	Inhibition
			8	Vardenafil	Inhibition
PDGFRA	PDGF-R-alpha	Receptor	1	Cediranib	Inhibition
			2	Imatinib	Inhibition
			3	Nintedanib	Inhibition
			4	Sunitinib	Inhibition
			5	XL999	Inhibition
IL6	IL-6	Receptor ligand	1	Aloperine	Inhibition
			2	Dexamethasone	Inhibition
			3	PF04236921	Inhibition
			4	Siltuximab	Inhibition
			5	Sirukumab	Inhibition
IL1B	IL-1 beta	Receptor ligand	1	Aloperine	Inhibition
			2	Canakinumab	Inhibition
			3	Doxycycline	Inhibition
			4	Gevokizumab	Inhibition
MMP1	MMP-1	Enzyme	1	Batimastat	Inhibition
			2	Doxycycline	Inhibition
			3	Marimastat	Inhibition
			4	Rebimastat	Inhibition
SRD5A2	S5AR2	Enzyme	1	Androstanolone	Inhibition
			2	Dutasteride	Inhibition
			3	Finasteride	Inhibition
			4	Izonsteride	Inhibition
PTGFR	PGF2aR	Receptor	1	(Z)-Unoprostone isopropyl	Activation
			2	Bimatoprost	Activation
			3	Travoprost	Activation

CSF2	GM-CSF	Receptor ligand	1	Bamirastine	Inhibition
			2	KB002	Inhibition
			3	MT203	Inhibition
IL1A	IL-1 alpha	Receptor ligand	1	Bamirastine	Inhibition
			2	Doxycycline	Inhibition
BDKRB2	BDKRB2	Receptor	1	Anatibant	Inhibition
			2	Labradimil	Activation
CTSS	CTSS	Enzyme	1	CRA028129	Inhibition
ETS1	ETS-1	Transcription factor	1	Estradiol	Inhibition
HGF	HGF	Receptor ligand	1	Rilotumumab	Inhibition
NFKB1	NFkB1 (p105)	Transcription factor	1	OT551	Inhibition
PIM1	Pim-1	Enzyme	1	SGI-1776	Inhibition
REL	c-Rel (NF-kB subunit)	Transcription factor	1	Apilimod	Inhibition
SAT1	SSAT	Enzyme	1	N(1),N(11)-Diethylnorspermine	Unspecified

Supplementary Table S3. Genes that encode direct targets for known drugs and are downregulated by IL-1β in HCAEC. Data are from 3 independent array experiments.

Gene symbol	Target	Type	Drug		Effect
HTR1D	HTR1D	Receptor	1	Almotriptan	Activation
			2	Dihydroergotamine	Activation
			3	Frovatriptan	Activation
			4	Ketanserin	Inhibition
			5	Naratriptan	Activation
			6	Rizatriptan	Activation
			7	Sumatriptan	Activation
			8	Ziprasidone	Unspecified
			9	Zolmitriptan	Activation
RARB	RARbeta	Transcription factor/nuclear receptor	1	Adapalene	Activation
			2	Etretinate	Activation
			3	Retinoic acid	Activation
			4	Tamibarotene	Activation
			5	Tazarotene	Activation
CXCR4	CXCR4	Receptor	1	Burixafor	Inhibition
			2	CTCE9908	Inhibition
			3	Plerixafor	Inhibition
FGFR2	FGFR2	Receptor	1	Cediranib	Inhibition
SCNN1A	alpha-ENaC	Sodium channel subunit	1	Amiloride	Inhibition

Supplementary Table S4. The targets of most significant pathways regulated by 4 h IL-1β treatment in HCAEC. Data are from 3 independent array experiments.

Targets	Regulation
C3[a, e, j]	Upregulated
CCL2[a, b, c, e, h]	Upregulated
CX3CL1[a]	Upregulated
ENA-78 (*CXCL5*)[b, c]	Upregulated
HGF[e]	Upregulated
ICAM1[a,c, d, e, f, g, h]	Upregulated
I-kB (*NFKBID*)[b,c, d, f, h, i]	Upregulated
IL-1 alpha[b,e, f, h]	Upregulated
IL-1 beta[a, b, c, e, f, g, h, i]	Upregulated
IL-6[a, b, c, d, e, f, g, h]	Upregulated
IL-8 [a, b, c, d, e, f, g, h]	Upregulated
A20 (*TNFAIP3*)[a]	Upregulated
C6[j]	Upregulated
CCL20[c]	Upregulated
CCL5[a, b, e, g]	Upregulated
CCL7[c]	Upregulated
c-IAP2 (*BIRC3*)[i]	Upregulated
COX-2 (*PTGS2*)[c, d, h]	Upregulated
CSF1[a, e]	Upregulated
E-selectin (*SELE*)[e, g, h]	Upregulated
ETS1[d]	Upregulated
F3[e, f, g]	Upregulated
Factor B (*CFB*)[j]	Upregulated
GCP2 (*CXCL6*)[c]	Upregulated
G-CSF (*CSF3*)[c]	Upregulated
GM-CSF (*CSF2*)[a, b, c, e, g]	Upregulated
GRO-1 (*CXCL1*)[a, c]	Upregulated
GRO-2 (*CXCL2*)[a]	Upregulated
GRO-3 (*CXCL3*)[a]	Upregulated
IL-18R1[h]	Upregulated
IL18RAP[h]	Upregulated
IP10 (*CXCL10*)[a, b]	Upregulated
IRAK2[i]	Upregulated
IRF1[a]	Upregulated
MDA-5 (*IFIH1*)[a]	Upregulated
MIP-1-alpha (*CCL3*)[a, b, f, g]	Upregulated
MIP-1-beta (*CCL4*)[b, f]	Upregulated
MMP-1[a, c, d]	Upregulated
NF-kB[a, c, d, e, f, g, h, i]	Upregulated
NF-kB p50/p50[b]	Upregulated
NF-kB1 (p105)[i]	Upregulated
NF-kB1 (p50)[b, i]	Upregulated
NFKBIA[d, f]	Upregulated
Pellino 1 (*PELI1*)[i]	Upregulated
PTX3[j]	Upregulated
RelB (NF-kB subunit)[i]	Upregulated

RIG-I (*DDX58*)[a]	Upregulated
TLR2[f, i]	Upregulated
TNF-alpha[a, b, d, e, f, g, h, i]	Upregulated
VCAM1[a, d, e, f, g, h]	Upregulated

[a]Regulated in 'Glomerular injury in Lupus Nephritis' pathway
[b]Regulated in 'PDE4 regulation of cyto/chemokine expression in arthritis' pathway
[c]Regulated in 'Immune response-IL-17 signaling pathways' pathway
[d]Regulated in 'Immune response-MIF-mediated glucocorticoid regulation' pathway
[e]Regulated in 'Vascular endothelial cell damage in SLE' pathway
[f]Regulated in 'Immune response_HMGB1/RAGE signaling pathway'
[g]Regulated in 'Substance P-mediated inflammation and pain in Sickle cell disease' pathway
[h]Regulated in 'Immune response_IL-18 signaling' pathway
[i]Regulated in 'Signal transduction_NF-kB activation pathways'
[j]Regulated in 'Immune response_Alternative complement pathway'

Supplementary Table S5. GO listing of disease states in which either HGF or IL-1β are involved.

Pathological states associated with only HGF	Pathological states associated with only IL-1β
Adenocarcinoma	Abortion, Habitual
Adenoma	Abortion, Spontaneous
Amyloidosis	Achlorhydria
Amyotrophic Lateral Sclerosis	Acute Kidney Injury
Astrocytoma	Aggressive Periodontitis
Breast Diseases	Alcoholism
Carcinoma, Lobular	Alopecia Areata
Carcinoma, Renal Cell	Alzheimer Disease
Carcinoma, Squamous Cell	Alzheimer disease, early onset
Carotid Artery Diseases	Alzheimer disease, late onset
Cerebral Infarction	Anemia
Cholesteatoma	Anemia, Sickle Cell
Cicatrix	Apnea
Diabetic Retinopathy	Appendicitis
Emphysema	Arterial Occlusive Diseases
Endometrial Neoplasms	Arthritis, Juvenile
Gingival Overgrowth	Arthritis, Psoriatic
Gliosis	Arthritis, Reactive
Head and Neck Neoplasms	Arthritis, Rheumatoid
Hearing Loss	Asbestosis
Hepatitis, Alcoholic	Aspergillosis
Hernia	Asthma
Hernia, Inguinal	Atrophy
Hyperplasia	Behcet Syndrome
Hypertrophy	Biliary Atresia
Hypopharyngeal Neoplasms	Bipolar Disorder
Infarction	Bone Resorption
Kidney Neoplasms	Brain Ischemia
Leukemia, Lymphocytic, Chronic, B-Cell	Bronchial Diseases
	Bronchopulmonary Dysplasia
Liver Neoplasms	Burning Mouth Syndrome
Lung Diseases	Cachexia
Lymphedema	Calculi
Meningioma	Carcinoma, Bronchogenic
Motor Neuron Disease	Carcinoma, Lewis Lung
Mouth Neoplasms	Cardiovascular Diseases
Moyamoya Disease	Celiac Disease
Myopia	Cholangiocarcinoma
Myopia, Degenerative	Cholangitis, Sclerosing
Nasopharyngeal Neoplasms	Chorioamnionitis
Neurilemmoma	Chronic Periodontitis
Neurofibromatosis 1	Coronary Artery Disease
Oligodendroglioma	Coronary Disease
Osteoarthritis	Coronary Restenosis
	Craniocerebral Trauma

Peripheral Nervous System-Neoplasms	Crohn Disease
	Cystic Fibrosis
Pituitary Neoplasms	Dementia
Pleural Neoplasms	Dengue
Pneumonia	Depressive Disorder, Major
Poisoning	Dermatitis
Pulmonary Embolism	Dermatitis, Allergic Contact
Pulmonary Fibrosis	Dermatitis, Atopic
Renal Insufficiency	Dermatitis, Contact
Retinal Neovascularization	Dermatitis, Irritant
Rhabdomyosarcoma	Dermatomyositis
Sarcoma, Alveolar Soft Part	Diabetes Mellitus
Sclerosis	Diabetes Mellitus, Type 1
Skin Neoplasms	Diabetes Mellitus, Type 2
Skin Ulcer	Diabetes, Gestational
Spinal Cord Neoplasms	Diabetic Nephropathies
Thromboembolism	Duodenal Ulcer
Thyroid Carcinoma, Papillary	Dyspepsia
Ulcer	Dysthymic Disorder
	Epilepsy
	Epilepsy, Temporal Lobe
	Esophageal Neoplasms
	Esophagitis
	Esophagitis, Peptic
	Familial Mediterranean Fever
	Fever
	Fibroma
	Gallstones
	Gastritis
	Gastritis, Atrophic
	Gastrointestinal Diseases
	Gingivitis
	Glaucoma
	Glaucoma, Open-Angle
	Glomerulonephritis, IGA
	Glossitis
	Glossitis, Benign Migratory
	Gout
	Graft vs Host Disease
	Graves Disease
	Hallucinations
	Heartburn
	Helicobacter Infections
	Hemophilia A
	Hemorrhage
	Hepatitis B
	Hepatitis B, Chronic
	Histiocytosis
	HIV Infections
	HTLV-I Infections

	Huntington Disease
	Hyperoxia
	Hypersensitivity, Immediate
	Hypothermia
	Hypoxia-Ischemia, Brain
	Inflammatory Breast Neoplasms
	Intervertebral Disc Displacement
	Intestinal Diseases
	Intestinal Neoplasms
	Jaundice
	Keratoconjunctivitis Sicca
	Keratoconus
	Kidney Failure, Chronic
	Leukemia, Megakaryoblastic, Acute
	Leukemoid Reaction
	Lichen Planus
	Lichen Planus, Oral
	Liver Cirrhosis, Alcoholic
	Liver Cirrhosis, Biliary
	Lupus Nephritis
	Lyme Disease
	Lymphoma, B-Cell, Marginal Zone
	Malaria
	Malaria, Cerebral
	Malaria, Falciparum
	Malnutrition
	Maxillary Neoplasms
	Meningococcal Infections
	Metabolic Syndrome X
	Mevalonate Kinase Deficiency
	Mitral Valve Prolapse
	Multiple Sclerosis
	Multiple Sclerosis, Chronic Progressive
	Multiple Sclerosis, Relapsing-Remitting
	Muscular Diseases
	Myelodysplastic Syndromes
	Nasal Polyps
	Neck Injuries
	Necrosis
	Nephrotic Syndrome
	Neuroblastoma
	Neurofibroma, Plexiform
	Ossification of Posterior Longitudinal Ligament
	Osteitis Deformans
	Osteoarthritis, Knee
	Osteonecrosis
	Osteoporosis, Postmenopausal
	Otitis
	Otitis Media

	Otitis Media with Effusion
	Overweight
	Pancreatitis
	Papillomavirus Infections
	Paraproteinemias
	Parkinson Disease
	Pemphigoid, Bullous
	Pemphigus
	Periodontitis
	Pneumoconiosis
	Premature Birth
	Prostatic Hyperplasia
	Psoriasis
	Purpura
	Purpura, Schoenlein-Henoch
	Purpura, Thrombocytopenic, Idiopathic
	Q Fever
	Raynaud Disease
	Respiratory Distress Syndrome, Adult
	Respiratory Tract Infections
	Retinoblastoma
	Rhinitis, Allergic, Perennial
	Root Resorption
	Sarcoidosis
	Schizophrenia
	Sepsis
	Severe Dengue
	Shock, Septic
	Silicosis
	Sinusitis
	Sjogren's Syndrome
	Sleep Apnea, Obstructive
	Spinal Osteophytosis
	Spondylitis, Ankylosing
	Stomach Diseases
	Stomach Ulcer
	Stomatitis
	Stomatitis, Aphthous
	Stroke
	Subarachnoid Hemorrhage
	Tenosynovitis
	Thyroid Diseases
	Thyroiditis, Subacute
	Tooth Diseases
	Tooth Loss
	Tuberculosis
	Tuberculosis, Pulmonary
	Tumor Virus Infections
	Uterine Neoplasms
	Vaginal Diseases

	Vaginosis, Bacterial<br>Vasculitis<br>Venous Thrombosis<br>Vulvar Neoplasms<br>Waldenstrom Macroglobulinemia<br>Zollinger-Ellison Syndrome

Supplementary Table S6. GO listing of disease states in which both HGF and IL-1β are involved.

Pathological states common where both IL-1β and HGF are involved
Alveolar Bone Loss
Arteriosclerosis
Arthritis
Bacterial Infections
Brain Injuries
Breast Neoplasms
Carcinoma
Carcinoma, Ductal, Breast
Carcinoma, Hepatocellular
Carcinoma, Non-Small-Cell Lung
Colitis, Ulcerative
Colonic Neoplasms
Colorectal Neoplasms
Dry Eye Syndromes
Endometriosis
Fibrosis
Glioblastoma
Glioma
Hepatitis
Hepatitis C
Hepatitis C, Chronic
Hepatitis, Chronic
Hypertension
Hypoxia
Idiopathic Pulmonary Fibrosis
Infection
Inflammation
Inflammatory Bowel Diseases
Insulin Resistance
Leukemia
Leukemia, Myeloid
Leukemia, Myeloid, Acute
Liver Cirrhosis
Lung Neoplasms
Lupus Erythematosus, Systemic
Lymphoma
Lymphoma, Non-Hodgkin
Medulloblastoma
Melanoma
Melanoma, Cutaneous Malignant
Melanoma, Uveal
Monoclonal Gammopathy of Undetermined Significance
Mucocutaneous Lymph Node Syndrome
Multiple Myeloma
Myocardial Infarction
Neoplasm Metastasis

Neoplasms
Obesity
Osteoporosis
Ovarian Neoplasms
Pancreatic Neoplasms
Periodontal Diseases
Prostatic Neoplasms
Pulmonary Disease, Chronic Obstructive
Rectal Neoplasms
Sarcoma
Sarcoma, Synovial
Scleroderma, Systemic
Small Cell Lung Carcinoma
Sprains and Strains
Stomach Neoplasms
Systemic Inflammatory Response Syndrome
Thrombosis
Thyroid Neoplasms
Urinary Bladder Neoplasms
Uterine Cervical Neoplasms
Wounds and Injuries

Supplementary Table S7. GO listing of pathways associated with HGF.

Pathway map	Map type
Cell adhesion_PLAU signaling	Normal
Colorectal cancer (general schema)	Pathology
Cytoskeleton remodeling_Hyaluronic acid/ CD44 signaling pathways	Normal
Development_Growth factors in regulation of oligodendrocyte precursor cell proliferation	Normal
Development_HGF signaling pathway	Normal
Development_HGF-dependent inhibition of TGF-beta-induced EMT	Normal
Development_PIP3 signaling in cardiac myocytes	Normal
Development_Regulation of epithelial-to-mesenchymal transition (EMT)	Normal
Development_Role of HGF in hematopoietic stem cell mobilization	Normal
Immune response_PGE2 signaling in immune response	Normal
Ovarian cancer (main signaling cascades)	Pathology
Resolution of inflammation in healing myocardial infarction	Pathology
Role of alpha-6/beta-4 integrins in carcinoma progression	Pathology
Role of growth factor receptors transactivation by Hyaluronic acid / CD44 signaling in tumor progression	Pathology
Transcription_Hypoxia- and receptor-mediated HIF-1 activation	Normal
Transport_Macropinocytosis regulation by growth factors	Normal
Vascular endothelial cell damage in SLE	Pathology
wtCFTR and deltaF508 traffic / Membrane expression (normal and CF)	Pathology

Supplementary Table S8. Targets involved in 'Lupus Erythematosus, Systemic' that were regulated by IL-1β in HCAEC as listed by Metacore GO biomarker assessment work flow centering immune system diseases. Data are from 3 independent array experiments.

Targets
ARID5B
BCL6
BCO2
BIRC3
C1R
C3
C5orf56
C6orf10
CAPS2
CCL2
CCL20
CCL3
CCL4
CCL5
CD5
CD69
CD70
CD83
CFB
CSF1
CSF2
CX3CL1
CXCL1
CXCL2
CXCL10
CXCR4
CYP1A1
DDX58
EBI3
EGFL7
ERC2
ETS1
F3
FCRL3
FOXJ1
FOXP3
HGF
ICAM1
ICOSLG
IFIH1
IFIT5
IFNGR2
IL15
IL16

IL18R1
IL1A
IL1B
IL6
IL7R
IL8
INHBA
ISG20
KIF6
MBP
MMP1
MS4A2
NAMPT
NFKB1
NFKBIA
NOD2
ODZ1
PARP14
PDLIM4
PTGS2
PTPRC
PTX3
RASGRP1
REL
RELB
RIPK2
SAMD9L
SELE
SERPINB2
SLC6A4
SOCS1
SOD2
STAT5A
TAP1
THBD
TIFA
TLR2
TNF
TNFAIP3
TNFRSF11B
TNFSF10
TNIP1
TRAF1
VCAM1

Supplementary Figure S1. The ten biological pathways most significantly (*P*<0.05) regulated by 4 h IL-1β treatment in HCAEC. Pathways represented as histograms are ranked by the –log value (*P* value). Data are from 3 independent experiments.

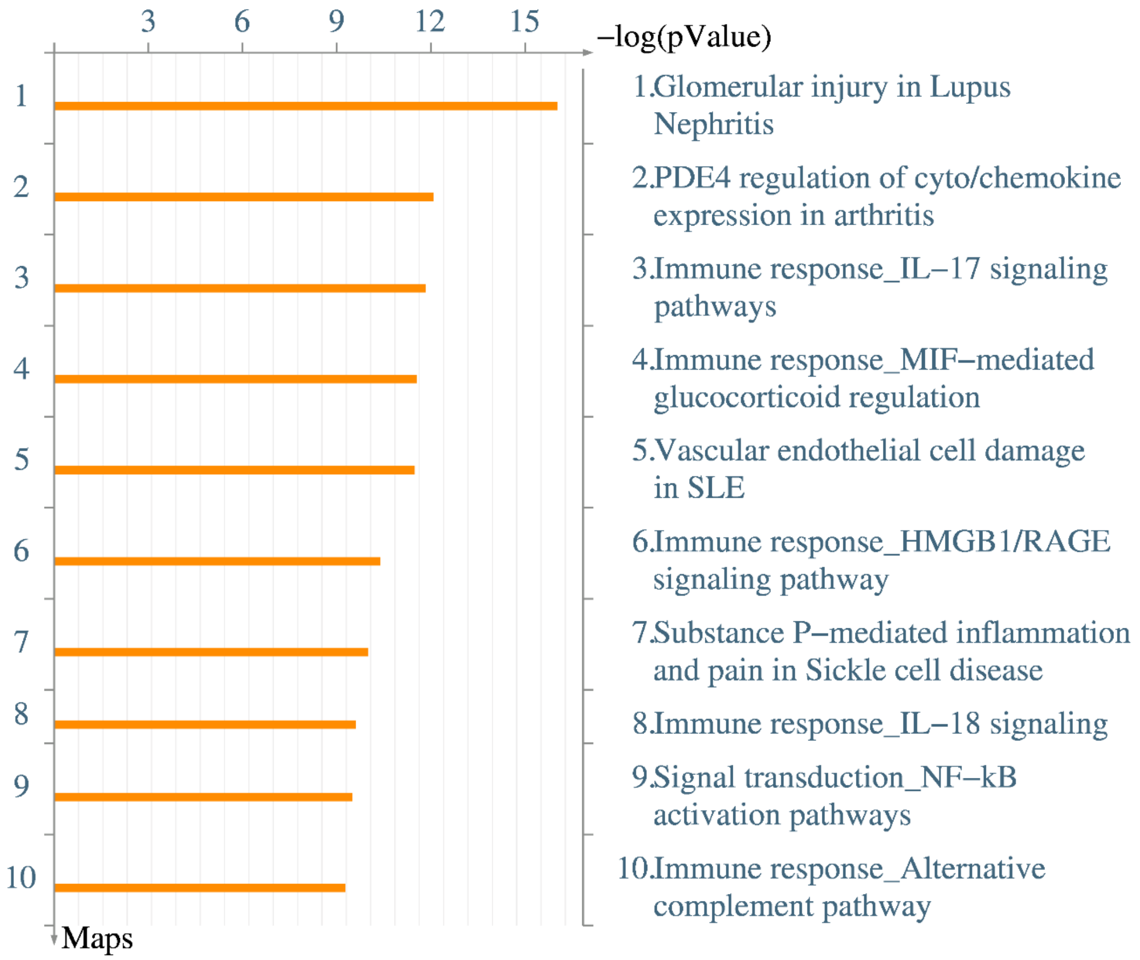

Supplementary Figure S2. Metacore™ GO biomarker assessment work flow centering immune system diseases showing the top 10 disease profiles enriched in 4 h IL-1β transcriptome. Diseases represented as histograms are ranked by the –log value (*P* value). Data are from 3 independent experiments.

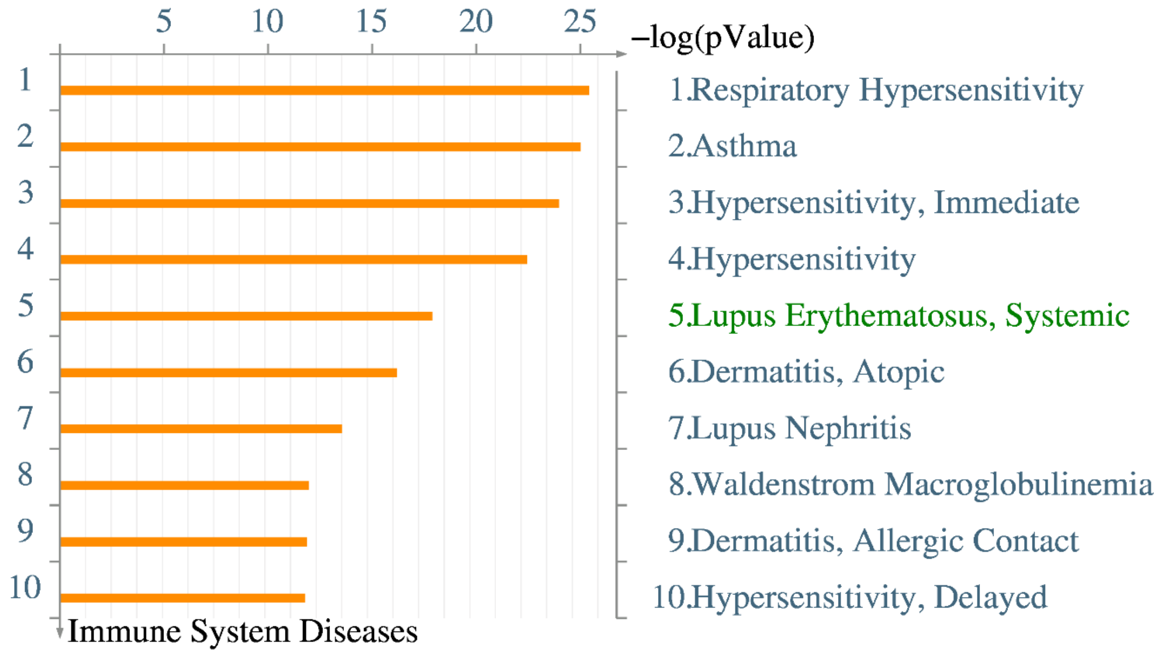

Supplement: Supplementary file 1 [file Data_Sheet_1.pdf]
